# Differentiated models of service delivery for antiretroviral treatment of HIV in sub-Saharan Africa: a rapid review protocol

**DOI:** 10.1186/s13643-019-1210-6

**Published:** 2019-12-06

**Authors:** Lawrence Long, Salome Kuchukhidze, Sophie Pascoe, Brooke Nichols, Refiloe Cele, Caroline Govathson, Amy Huber, David Flynn, Sydney Rosen

**Affiliations:** 1 Department of Global Health, Boston University, Boston, USA; 2 Health Economics and Epidemiology Research Office, Department of Internal Medicine, School of Clinical Medicine, Faculty of Health Sciences, University of the Witwatersrand, Johannesburg, South Africa; 3 Alumni Medical Library, Boston University, Boston, USA

**Keywords:** Sub-Saharan Africa, HIV, differentiated care, Coverage, Effectiveness, Cost, Treatment

## Abstract

**Background:**

To meet global targets for the treatment of HIV, high-prevalence countries are launching or expanding differentiated models of service delivery (DSD) for antiretroviral therapy (ART). Ongoing studies report on metrics specific to individual models of care, but little is known about the overall scale, impact, costs, and benefits of widespread implementation of DSD. We will conduct a rapid review of recent literature on DSD currently in use in sub-Saharan Africa and identify gaps in the literature with respect to the description of delivery models, coverage, effectiveness, and cost.

**Methods:**

We will use an adapted version of the preferred reporting items for systematic reviews and meta-analysis protocols (PRISMA-P) for reporting. To avoid repeating earlier reviews, only sources reporting data on interventions conducted and/or patients starting antiretroviral treatment since 1 January 2016 will be included. Other inclusion criteria: must report on HIV-positive patients receiving antiretroviral therapy (ART) for the treatment of HIV/AIDS in sub-Saharan Africa; must describe an antiretroviral care intervention and identify the location, visit frequency, provider, patient group, and intervention intensity; and must report at least one of the following outcomes: population coverage, intervention uptake, treatment outcomes, cost or resource allocation, acceptability, or feasibility. Exclusion criteria: receiving ART as part of prevention of mother-to-child transmission (PMTCT) program or receiving preventive ART (PEP or PrEP). This review will include peer-reviewed articles and conference abstracts. Publication databases to be searched include PubMed, EMBASE, and Web of Science. For analysis, where possible, we will group the DSD by key characteristics (e.g., population served, visit frequency, visit location) and then report means and/or medians of coverage and outcomes with confidence intervals or IQRs. We will also descriptively compare and contrast different models of care, implementation challenges, and other non-quantitative information.

**Discussion:**

This review will provide an initial picture of the status quo for the implementation of DSD in sub-Saharan Africa and identify directions for research and implementation support in the future. This big-picture analysis will be useful for ministries of health, implementers, and donor agencies to inform decision-making on DSD scale-up.

**Systematic review registration:**

PROSPERO: CRD42019118230

## Background

As the availability of antiretroviral therapy (ART) for the treatment of HIV/AIDS has increased in resource-limited settings, there has been a move to develop and implement alternative treatment delivery models (also referred to as “differentiated models of service delivery” or DSD) in high prevalence countries in order to meet the global targets for HIV treatment while maintaining quality of care [1]. Alternative treatment delivery models typically differ across one or more of the service characteristics (provider, location, frequency, and intensity of care) and aim to provide a more patient-centric service [2]. Proponents of DSD also believe that alternative models utilize resources more efficiently, without compromising patient care [3, 4]. It is this promise of greater efficiency in a climate of constrained resources and increasing demand that is driving the rapid expansion of DSD [5–7].

Sub-Saharan Africa, with both a high burden of HIV and limited domestic public health resources, has been at the forefront of innovative, alternative treatment delivery models for the last decade. Most efforts focused on “stable” (virally suppressed) ART patients who are thought to require less medical oversight than those just starting treatment or failing therapy. These stable patients were placed in alternative models that incorporated some combination of fewer clinic visits, community service delivery, and interim monitoring by non-clinicians [8, 9]. Under increasing pressure to reach the UNAIDS “90-90-90” and now “95-95-95” goals, the use of alternative treatment delivery models grew rapidly to cover the full treatment continuum, increasingly including unstable patients [3, 10–13]. These models, both formal and informal, have now found their way into national HIV treatment strategies, with most high prevalence countries implementing one or more alternative models for HIV patients [14–16].

While a few of the early alternative models, such as adherence clubs and community adherence groups (CAG), have been described and evaluated in the literature, most studies were limited to clinical trials and small-scale implementation [17, 18]. In most instances, routine implementation was not matched with an evaluation or monitoring strategy designed to measure progress or impact. As a result, there are almost no routine care databases that document these models or reports on their effectiveness in generating the expected benefits.

As each country in sub-Saharan Africa develops and scales up its versions of service delivery models, global and national policy-makers, funders, and program managers are grappling with questions of current coverage and uptake, which models are most effective for which patients, whether DSD allows reallocation of “saved” resources, and how to sustain service delivery outside the clinic once donor support is no longer available. To assess what is already known about the implementation and outcomes of DSD, we will conduct a rapid systematic review of publications from the most recent 4 years (2016–2019) that describe and report on the most recent ART delivery models.

The objective of this rapid review is to summarize the most recent information available about differentiated models of antiretroviral treatment delivery currently in use in sub-Saharan Africa and identify gaps in the existing published literature and conference proceedings with respect to the description of ART delivery models, coverage, effectiveness, and cost. Approaches to delivering HIV treatment are changing rapidly, making it important that new evaluations focus on recent data. To ensure that our results come as close as possible to reflect the current situation, and to avoid repeating the efforts of previous reviews [5, 19–23], this rapid review is limited to data generated in 2016 or later .

## Methods

### Protocol and registration

We will conduct this rapid review following the definition and methodology outlined by the World Health Organization [24]. The protocol was registered on the International prospective register of systematic reviews (PROSPERO), further details are provided in Additional file 1. We will use an adapted version of the preferred reporting items for systematic reviews and meta-analysis protocols (PRISMA-P) for reporting this protocol as there is no currently accepted standardized reporting format for rapid reviews. The adapted PRISMA-P checklist is provided in Additional file 2 [25, 26].

### Inclusion/exclusion criteria

Publications and reports will be selected for inclusion in the review using the criteria shown in Table 1.

**Table 1:** Inclusion and exclusion criteria

Criterion	Include	Exclude
A. Population	• All ages • All genders • Confirmed HIV positive status • All risk groups (general, priority, key) • On any line of lifelong antiretroviral treatment (i.e., first, second, or third-line) • In sub-Saharan Africa	• Pregnant women in PMTCT programs • On ART for prevention (PEP or PrEP)
B. Intervention	• Delivery of lifelong ART that differs from standard or traditional care in terms of population, location, frequency, provider cadre, or services provided.	Report about a solely standard or traditional model for delivering ART, prior to any differentiation based on population, location, frequency, provider cadre, or services provided
C. Required descriptive data about model	Reports all of • Location—is care provided in the clinic, on the clinic campus, in the community or workplace, at home? • Frequency—how often does the patient interact with the healthcare system for each type of service (drug pickup, medical consultation)? • Provider—which cadre of clinical or lay staff provides the service? For example, nurses conduct the medical visits, while “expert patients” deliver drugs to the patient’s house. • Patient type and line (stable, newly initiated, not stable; first, second, or third line) • Services provided (visit intensity)—what occurs at each visit or interaction? Does visit include concomitant care or medication delivery for co-morbidities?	Description provided does not describe all the characteristics needed to define the model
D. Comparator	Not required—single-arm evaluations are eligible	None
E. Outcomes	Reports one or more of the following outcomes: • Coverage of population in need • Uptake by patients • Clinical outcome (e.g. retention in care, viral suppression) • Cost or resource allocation • Acceptability to patients or providers • Feasibility to implement	Insufficient detail provided to estimate at least one outcome
F. Timing	A majority of follow up data report on the delivery of antiretroviral treatment occurring in January 2016 or later	A majority of follow-up data report on the period before January 2016
G. Sector	Services provided to the public sector through the government managed public health infrastructure or through partner/NGO/private programs or facilities that serve the uninsured sector	Services or programs for privately (commercially) insured patients
H. Study design	Reports empirical data from retrospective or prospective cohort, including: • Randomized controlled trials • Observational studies (including single-arm evaluations) • Pre/post studies with or without a comparison group	• Systematic or other reviews • Case series or reports • Treatment guidelines • Mathematical models • Editorials • Commentaries

### Publication type and search strategy

This review will include peer-reviewed journal articles and peer-reviewed conference abstracts. The primary search strategy (see Additional file 3), which was developed and refined by two of the investigators (LL and SR) and a medical librarian (DF), will focus on established electronic databases, specifically PubMed, Embase, and Web of Science. The initial search string utilized MeSH terms to describe publications which reported on HIV treatment models meeting the relevant eligibility criteria. These MeSH terms were then expanded to include the entry terms to ensure recent publications not yet categorized with MeSH terms are captured. This initial search string and its results were reviewed by two of the investigators (LL and SR) to see if it could be further refined and a final PubMed search string was created by a medical librarian (DF). The PubMed search string was then transcribed into a search string for Embase and Web of Science by a medical librarian (DF). The secondary search strategy will include relevant conference websites for the years 2016-2019 (CROI, IAS, SAAIDS, SAHIVSOC, EACS, APACC, INTEREST, ZHRC, and ICASA—see Additional file 3 for full titles of conferences). This will be supplemented by manually reviewing the reference lists of included publications. The specific search strings for each database are noted in Additional file 3. Both the primary and secondary search will review articles with a publication date 1 January 2016 or later.

### Data and analysis

#### Record selection

Records generated by the search will be managed using Endnote ™ Version X9, Rayyan QCRI ^TM^, and Mendeley ^TM^ Version 1.19.2 software. Titles and abstracts from a sample of 50 records from the final citation library (with duplicates removed) will be reviewed by the full study team (LL, SR, SP, SK, BN, CG, RC) against the stated inclusion and exclusion criteria. The search criteria will then be clarified as needed. After this calibration process, the first review of all titles and abstracts will be conducted by three independent reviewers (SK, CG, RC) using the Rayyan ™ platform and will be blinded until all reviewers have completed the process. For publications for which reviewers have conflicting opinions, two study team members will assess reasons for exclusion and make a final determination. The remaining publications will be included in the second review. All publications included for the second review will be retrieved in full and assessed against the specified inclusion and exclusion criteria. The second review will be done by at least two reviewers (SK, CG) and any disagreement between reviewers will be resolved through discussion and review by a third independent reviewer (LL). The results of the search will be presented in a PRISMA flow diagram.

#### Data extraction

Data to be extracted from each publication included in the review are listed in Table 2.

**Table 2:** Fields to be extracted from selected publications

Category	Fields
Publication identifiers	Authors
	Article title
	Publication type (journal, abstract)
	Publication date
	Journal title with volume, issue, pages
Study design and site(s)	Design (cross-sectional, longitudinal, trial, etc.)
	Dates of data collection
	Names and locations of study sites
Population and participants	Age group (adults, adolescents, children)
	Risk group (general adults, people who inject drugs, men who have sex with men, transgender people, sex workers, health care workers)
	Sample size per arm
	Gender (% female)
	Year(s) of enrollment
	Duration of follow up (months)
Intervention	Location of service delivery
	Frequency of interaction
	Health care provider cadres
	ART regimen/line
	Services provided (intensity)
	Sector
Outcomes	Uptake (value, unit, detail)
	Cost (value, unit, detail)
	Treatment outcome (outcome type, detail/definition, value, unit, effect size, confidence interval)
	Acceptability
	Feasibility

A data extraction tool will be created in Excel™ and piloted on a subset (*n* = 5) of studies to determine whether fields can incorporate dropdown lists or other data validation checks to improve consistency in the data extraction. After refining the tool based on the pilot assessment, one reviewer (SK) will be responsible for extracting the data from the paper and a second reviewer will ensure data quality (CG). If the reviewers are unsure about the availability or suitability of data points this will be referred to a third reviewer (LL) for adjudication.

#### Data synthesis and analysis

We anticipate that the review will include a wide range of study designs and outcomes. We will start by reporting each study’s outcomes, by category (e.g., clinical outcomes, costs). Where possible, we will pool outcomes and report average and/or median values, with associated confidence intervals. Given the broad scope of the review and the attempt to be as inclusive as possible, however, it is unlikely that a formal statistical meta-analysis will be possible. Instead, we will describe the available data using basic descriptive statistics and narrative synthesis. If interventions appear similar to one another using the indicators listed in Table 1, we will also group results by intervention type. Similarly, where possible we will group and comment upon other common features (e.g., rural vs urban models of care).

#### Assessing the risk of bias

The risk of bias will be assessed using the Newcastle-Ottawa scale [27] recommended by the Cochrane Collaboration group [28] for non-randomized studies and the revised Cochrane Risk of Bias Tool for randomized controlled trials [29, 30]. No studies will be excluded based on the risk of bias assessment, but the score will be reported for each study included in the final report.

#### Amendments to the protocol

Significant amendments to the protocol after publication will be documented and reported in the results of the review.

## Discussion

The rapid review described in this protocol will synthesize current evidence related to differentiated models of antiretroviral treatment delivery being implemented in sub-Saharan Africa, providing an up-to-date evidence baseline for policy-makers, funders, and implementers. Depending on the results of the review, we anticipate that it will both inform these audiences about current progress and identify gaps in the evidence base requiring further research or evaluation.

## Supplementary information

**Additional file 1.** Registration in PROSPERO. https://www.crd.york.ac.uk/PROSPERO/display_record.php?RecordID=118230.

**Additional file 2.** Reporting standards—PRISMA-P Checklist.

**Additional file 3.** Database search strings.

## Data Availability

Not applicable—no datasets currently available.

## Abbreviations

AIDS: Acquired immune deficiency syndrome
ART: Antiretroviral therapy
CAG: Community adherence group
CommART: Community-Centred Models of ART Service Delivery
CQUIN: Coverage, Quality, and Impact Network
CROI: *Conference* on Retroviruses and Opportunistic Infections
EQUIP: Evaluation and Quality Improvement Program
HIV: Human immunodeficiency virus
IAS: International AIDS Society
PEPFAR: President’s Emergency Plan for AIDS Relief
PMTCT: Prevention of mother-to-child transmission
PRISMA-P: Preferred Reporting Items for Systematic reviews and Meta-Analysis Protocols
USAID DEC: United States Agency for International Development Experience Clearinghouse
