# Supplementary material for: Differentiated models of service delivery for antiretroviral treatment of HIV in sub-Saharan Africa: a rapid review protocol
Source: Syst Rev. 2019 Dec 6;8:314. doi: 10.1186/s13643-019-1210-6 (PMC6896778; doi:10.1186/s13643-019-1210-6)
Supplement: Supplementary file 1 — Additional file 1. Registration in PROSPERO. https://www.crd.york.ac.uk/PROSPERO/display_record.php?RecordID=118230. [file 13643_2019_1210_MOESM1_ESM.docx]

**Additional file 1 Registration in PROSPERO**

https://www.crd.york.ac.uk/PROSPERO/display_record.php?RecordID=118230
