# Supplementary material for: Differentiated models of service delivery for antiretroviral treatment of HIV in sub-Saharan Africa: a rapid review protocol
Source: Syst Rev. 2019 Dec 6;8:314. doi: 10.1186/s13643-019-1210-6 (PMC6896778; doi:10.1186/s13643-019-1210-6)
Supplement: Supplementary file 3 — Additional file 3. Database search strings. [file 13643_2019_1210_MOESM3_ESM.docx]

**Additional file 3 Database search strings**

| **Web of science** | |
| --- | --- |
|  | Search String |
| #1 | TS=(Africa South of the Sahara OR Sub-Saharan Africa OR Subsaharan Africa OR Africa, Sub- Saharan) |
| #2 | \|  \| TS=(HIV Seropositiv* OR Seropositiv* HIV OR Seropositiv*, HIV OR AIDS Seropositiv* OR Seropositiv*, AIDS OR Anti-HIV Positiv* OR Anti HIV Positiv* OR HTLV-III Seropositiv* OR HTLV III Seropositiv* OR HIV Seroconversion* OR Seroconversion*, HIV OR HTLV-III Seroconversion* OR HTLV III Seroconversion* OR Seroconversion*, HTLV-III OR AIDS Seroconversion* OR Seroconversion*, AIDS OR HIV Antibody Positivity OR Antibody Positivities, HIV OR Antibody Positivity, HIV OR HIV Antibody Positivities OR Positivities, HIV Antibody OR Positivity, HIV Antibody OR HIV Infections OR HIV Infection OR Infection, HIV OR Infections, HIV OR HTLV-III-LAV Infections OR HTLV III LAV Infections OR HTLV-III-LAV Infection OR Infection, HTLV-III-LAV OR Infections, HTLV-III-LAV OR T-Lymphotropic Virus Type III Infections, Human OR T Lymphotropic Virus Type III Infections, Human OR HTLV-III Infections OR HTLV III Infections OR HTLV-III Infection OR Infection, HTLV-III OR Infections, HTLV-III OR HIV Coinfection OR Coinfection, HIV OR Coinfections, HIV OR HIV Coinfections) \| \| --- \| --- \| |
| #3 | TS=(Anti-HIV Agents OR Agents, Anti-HIV OR Anti HIV Agents OR Anti-AIDS Agents OR Agents, Anti-AIDS OR Anti AIDS Agents OR Anti-HIV Drugs OR Anti HIV Drugs OR Drugs, Anti-HIV OR AIDS Drugs OR Drugs, AIDS OR Anti-AIDS Drugs OR Anti AIDS Drugs OR Drugs, Anti-AIDS OR antiretroviral therap* OR Antiretroviral Therap*, Highly Active OR Highly Active Antiretroviral Therapy OR HAART) |
| #4 | \|  \| #3 AND #2 AND #1 \| \| --- \| --- \| |
| #5 | \|  \| TS=(Drug Evaluation OR Drug Evaluations OR Evaluation, Drug OR Evaluations, Drug OR Evaluation Studies, Drug OR Drug Evaluation Studies OR Drug Evaluation Study OR Evaluation Study, Drug OR Studies, Drug Evaluation OR Study, Drug Evaluation OR Drug Approval OR Approval, Drug OR Approvals, Drug OR Drug Approvals OR Food and Drug Administration Drug Approval OR Drug Approval Process OR Approval Process, Drug OR Approval Processes, Drug OR Drug Approval Processes OR Process, Drug Approval OR Processes, Drug Approval OR New Drug Approval Process OR New Drug Approval OR Approval, New Drug OR Approvals, New Drug OR Drug Approval, New OR Drug Approvals, New OR New Drug Approvals OR Food and Drug Administration Drug Approval Process OR "Clinical Trials" OR "Clinical Trial") \| \| --- \| --- \| |
| #6 | #4 NOT #5 |
| #7 | #4 NOT #5 Timespan=2016-2018 |
| **Embase** | |
| #1 | 'africa south of the sahara'/exp OR 'africa south of the sahara' OR 'black africa' OR 'sub saharan africa' OR 'subsaharan africa' |
| #2 | 'human immunodeficiency virus infection'/exp OR 'hiv infection' OR 'hiv infections' OR 'human immunodeficiency virus infection' OR 'hiv seropositivity' OR 'human immunodeficiency virus encephalopathy' |
| #3 | 'anti human immunodeficiency virus agent'/exp OR 'anti hiv agent' OR 'anti hiv agents' OR 'anti human immunodeficiency virus agent' OR 'anti-hiv agents' OR 'highly active antiretroviral therapy'/exp OR 'haart' OR 'antiretroviral therapy, highly active' OR 'highly active antiretroviral theraphy' |
| #4 | 'drug screening'/exp OR 'assay, subrenal capsule' OR 'drug evaluation' OR 'drug scanning' OR 'drug screening' OR 'drug screening assays, antitumor' OR 'drug screening assays, antitumour' OR 'drug testing' OR 'drug trial' OR 'pharmaceutical screening' OR 'screening, drug' OR 'testing, drug' OR 'tumour stem cell assay' OR 'xenograft model antitumor assays' OR 'xenograft model antitumour assays' |
| #5 | #1 AND #2 AND #3 |
| #6 | #4 NOT #5 |
| #7 | #5 NOT #4 |
| #8 | #5 NOT #4 AND [2016-2018]/py |
| **Pubmed** | |
|  | (((("Africa South of the Sahara"[Mesh] OR Sub-Saharan Africa OR Subsaharan Africa OR Africa, Sub-Saharan)) AND ((((("HIV Seropositivity"[Mesh] OR HIV Seropositivities OR Seropositivities, HIV OR Seropositivity, HIV OR AIDS Seropositivity OR AIDS Seropositivities OR Seropositivities, AIDS OR Seropositivity, AIDS OR Anti-HIV Positivity OR Anti HIV Positivity OR Anti-HIV Positivities OR Positivities, Anti-HIV OR Positivity, Anti-HIV OR HTLV-III Seropositivity OR HTLV III Seropositivity OR HTLV-III Seropositivities OR Seropositivities, HTLV-III OR Seropositivity, HTLV-III OR HIV Seroconversion OR HIV Seroconversions OR Seroconversion, HIV OR Seroconversions, HIV OR HTLV-III Seroconversion OR HTLV III Seroconversion OR HTLV-III Seroconversions OR Seroconversion, HTLV-III OR Seroconversions, HTLV-III OR AIDS Seroconversion OR AIDS Seroconversions OR Seroconversion, AIDS OR Seroconversions, AIDS OR HIV Antibody Positivity OR Antibody Positivities, HIV OR Antibody Positivity, HIV OR HIV Antibody Positivities OR Positivities, HIV Antibody OR Positivity, HIV Antibody)) OR ("HIV Infections" [Mesh] OR HIV Infection OR Infection, HIV OR Infections, HIV OR HTLV-III-LAV Infections OR HTLV III LAV Infections OR HTLV-III-LAV Infection OR Infection, HTLV-III-LAV OR Infections, HTLV-III-LAV OR T-Lymphotropic Virus Type III Infections, Human OR T Lymphotropic Virus Type III Infections, Human OR HTLV-III Infections OR HTLV III Infections OR HTLV-III Infection OR Infection, HTLV-III OR Infections, HTLV-III OR HIV Coinfection OR Coinfection, HIV OR Coinfections, HIV OR HIV Coinfections))) AND ((("Anti-HIV Agents"[Mesh] OR Agents, Anti-HIV OR Anti-HIV Agents OR Anti-AIDS Agents OR Agents, Anti-AIDS OR Anti AIDS Agents OR Anti-HIV Drugs OR Anti HIV Drugs OR Drugs, Anti-HIV OR AIDS Drugs OR Drugs, AIDS OR Anti-AIDS Drugs OR Anti AIDS Drugs OR Drugs, Anti-AIDS OR "antiretroviral therapy"[TIAB])) OR ("Antiretroviral Therapy, Highly Active"[Mesh] OR Highly Active Antiretroviral Therapy OR HAART))))) NOT (((("Drug Evaluation"[Mesh] OR Drug Evaluations OR Evaluation, Drug OR Evaluations, Drug OR Evaluation Studies, Drug OR Drug Evaluation Studies OR Drug Evaluation Study OR Evaluation Study, Drug OR Studies, Drug Evaluation OR Study, Drug Evaluation)) OR ("Drug Approval"[Mesh] OR Approval, Drug OR Approvals, Drug OR Drug Approvals OR Food and Drug Administration Drug Approval OR Drug Approval Process OR Approval Process, Drug OR Approval Processes, Drug OR Drug Approval Processes OR Process, Drug Approval OR Processes, Drug Approval OR New Drug Approval Process OR New Drug Approval OR Approval, New Drug OR Approvals, New Drug OR Drug Approval, New OR Drug Approvals, New OR New Drug Approvals OR Food and Drug Administration Drug Approval Process)) OR ("Clinical Trials as Topic"[Mesh] OR Clinical Trial as Topic)) |

**Conference Abstract Databases / Books Reviewed**

- International AIDS Society (IAS) 2016,2017,2018
- Conference on Retroviruses and Opportunistic Infections (CROI) 2016,2017,2018, 2019
- South African AIDS Conference (SAAIDS) 2017
- Southern African HIV Clinicians Society (SAHIVSOC) 2016,2018
- European AIDS Conference (EACS) 2017
- INTEREST Conference 2016,2017,2018
- Zambia Health Research Conference (ZHRC) 2018
- Asia Pacific AIDS & Co-infections Conference (APACC) 2016, 2017
- International Conference on AIDS and STI’s in Africa (ICASA) 2017
